# An Unusual Case of Anterior Elbow Dislocation Without Bony Injury

**DOI:** 10.7759/cureus.13655

**Published:** 2021-03-02

**Authors:** Eoin Fahey, Mohammad Gaafar, Catherine Bossut

**Affiliations:** 1 Orthopaedics, St. James's Hospital, Dublin, IRL

**Keywords:** anterior elbow dislocation, instability, open reduction

## Abstract

While elbow dislocation is a common occurrence, the vast majority of them dislocate posteriorly and are due to disruption of the elbow stabilizers, which start on the lateral side and proceed medially, disrupting the anterior and posterior stabilizing structures. We present an unusual case of anterior elbow dislocation, with disruption of the medial stabilizing structures and anterior capsule, without any bony injury.

A 44-year-old man presented to the ED after being assaulted. While the exact mechanism of injury was unclear, the patient believes he had been struck with a heavy object on the posterior aspect of his elbow. His dislocation was reduced in the ED, but was highly unstable after reduction. Further imaging revealed disruption of his medial collateral ligaments and common flexor origin. He went on to have an open repair of his medial structures with suture anchors. After six weeks of follow-up he was doing well, with no further episodes of instability and a good functional range of movement.

Though rare, anterior elbow dislocations have been reported sporadically in the literature. Surgeons and ED doctors dealing with these injuries should be aware that the maneuvers to relocate the elbow will be different compared to the standard maneuvers used for posterior dislocations. Patients should be examined for stability after reduction, especially on valgus stressing. We would advocate for low threshold for performing an examination under anesthesia (EUA), with open repair of the stabilizing structures if persistently unstable after reduction.

## Introduction

Elbow dislocation is commonly quoted as the second most frequently dislocated joint [1], with an incidence of 5.21 elbow dislocations per 100,000 patient years [2]. The vast majority of these dislocations occur posteriorly with anterior elbow dislocation being relatively rare. Anterior dislocation without periarticular fracture is extremely unusual and has only been reported sporadically in the literature. Loss of range of movement is a common problem after elbow dislocation [3]. In posterior dislocations it has been shown that better range of movement is achieved the earlier patients are mobilized [4]. The case we report here adds to the reported literature on a rare injury pattern. It also demonstrates the treatment technique we used to allow early mobilization.

## Case presentation

A 44-year-old man attended the ED with pain and deformity of left elbow. He gave a history of having been assaulted by a group of people during a street protest. He recalls being struck by a heavy object on the posterior aspect of a flexed elbow as someone was attempting to pull him away from the situation by the arm. This was an isolated injury. There was some paraesthesia in the median nerve distribution initially, but this settled after reduction. He had no sensory or motor deficits of the median, radial or ulnar nerves.

He had no significant medical history, and had no personal or family history of connective tissue disorders or hypermobility.

**Figure 1:**
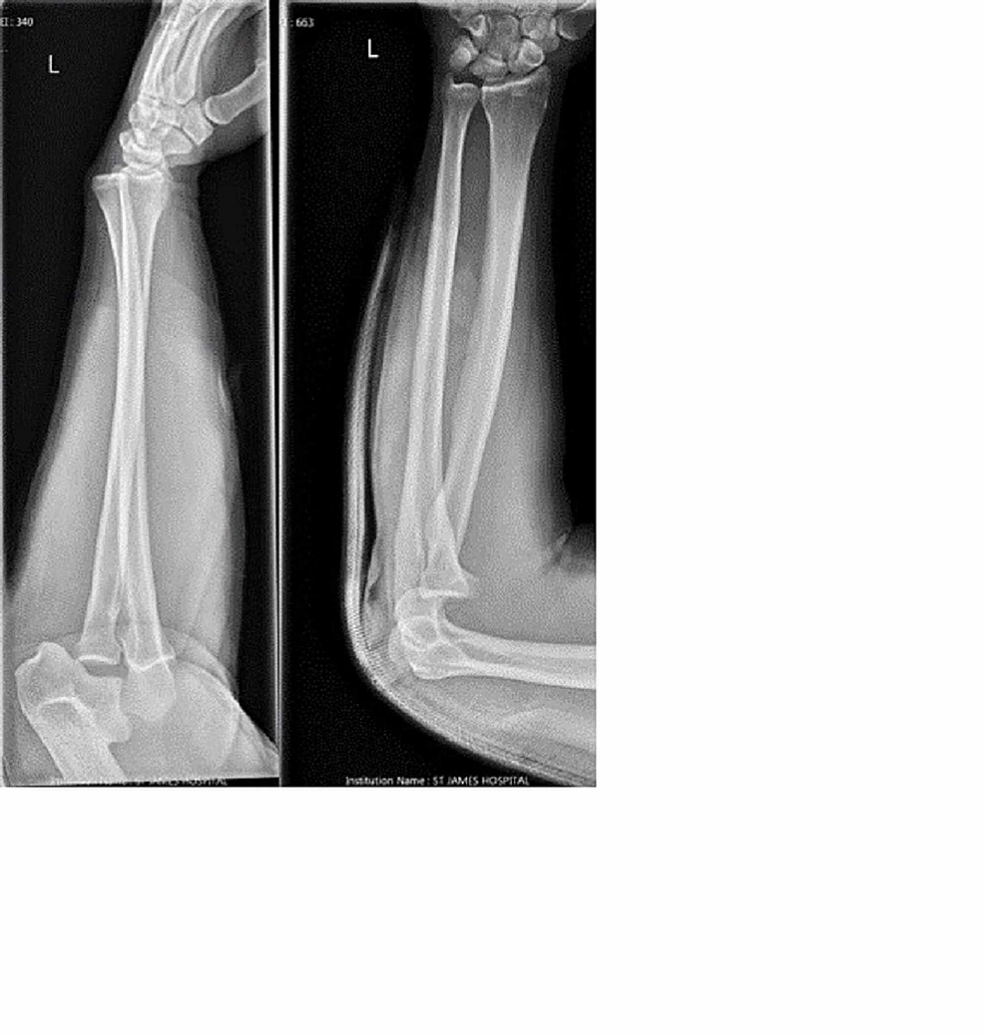
Images taken in ED on presentation.

A radiograph was performed on presentation (Figure 1). This showed an anterior dislocation of the left elbow, with no obvious periarticular fractures. The dislocation was reduced under sedation in the ED. The maneuver used to achieve this reduction was applying traction at the flexed elbow while an assistant held the humerus stable, before posteriorly translating the forearm and flexing the elbow. There was no obvious clunk during reduction, but the clinical deformity resolved. He was placed in an above elbow backslab in a flexed position and X-ray confirmed that the elbow was reduced. He was admitted under the orthopedic team while CT and MRI were being arranged. 

On Day 1 postadmission the patient started to complain of increasing pain, and his elbow was found to have re-dislocated while being in the backslab. At this stage he underwent closed reduction with the aid of a full general anesthetic and muscle relaxant, as well as an examination under anesthesia (EUA). During EUA the elbow was found to be highly unstable and dislocated if extended past 100 degrees. The elbow was very unstable on valgus stressing, with no firm end point of medial collateral ligaments. The patient was placed in an above elbow cast, flexed to 80 degrees, until further imaging could be carried out.

**Figure 2:**
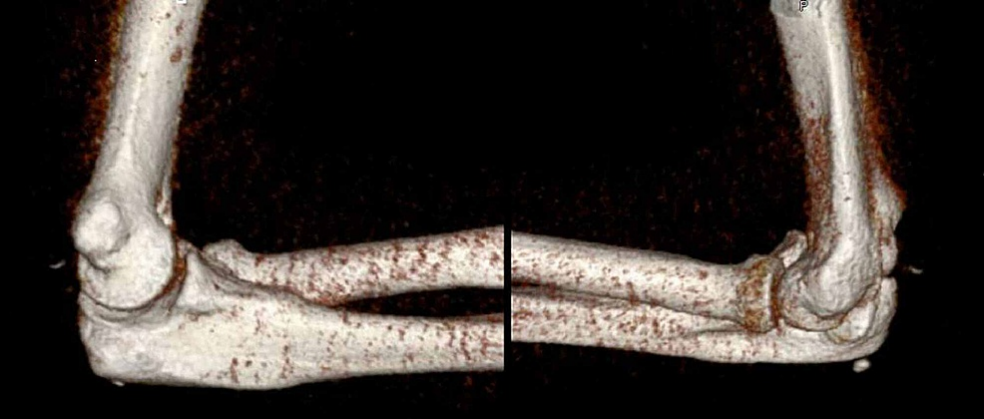
3D reconstruction of the elbow from the CT scan. 3D, three-dimensional

CT and MRI of the elbow were carried out. A 3D reconstruction after relocation is shown in Figure 2. This shows an anatomical alignment of the elbow joint after reduction, with some tiny avulsion fragments posteriorly, likely from the trochlea or the olecranon. The MRI reported “At least partial disruption of the ulnar collateral ligament” and “High partial tear of the common flexor origin”. The patient was kept in cast until definitive surgery could be carried out.

**Figure 3:**
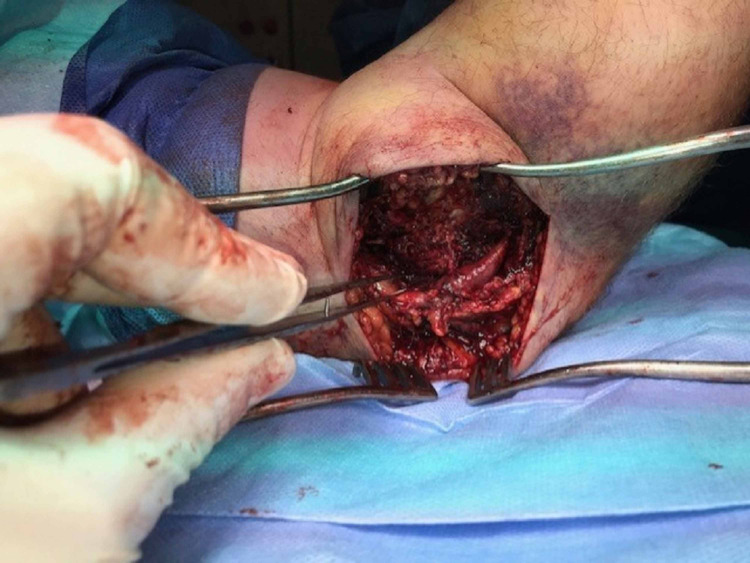
Bruising and edema observed around the ulnar nerve.

A medial (flexor carpi ulnaris splitting) approach to the elbow was carried out. The intraoperative findings were as follows: 1) complete avulsion of the common flexor origin, 2) complete avulsion of the ulnar collateral ligaments (UCL) from the medial epicondyle, 3) incarceration of the anterior joint capsule between the trochlea and the ulna, and 4) extensive bruising and edema of the ulnar nerve (Figure 3).

**Figure 4:**
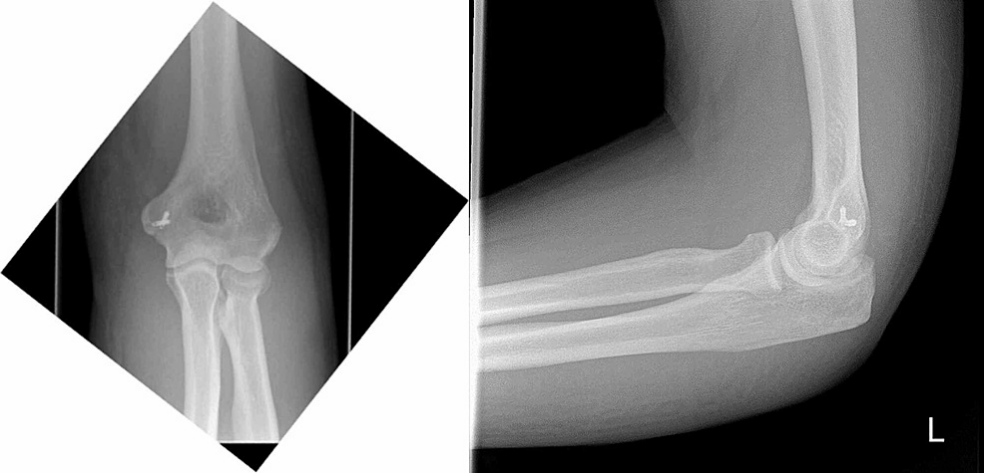
AP and lateral radiographs of elbow postoperatively demonstrating the position of suture anchors. AP, anteroposterior

**Figure 5:**
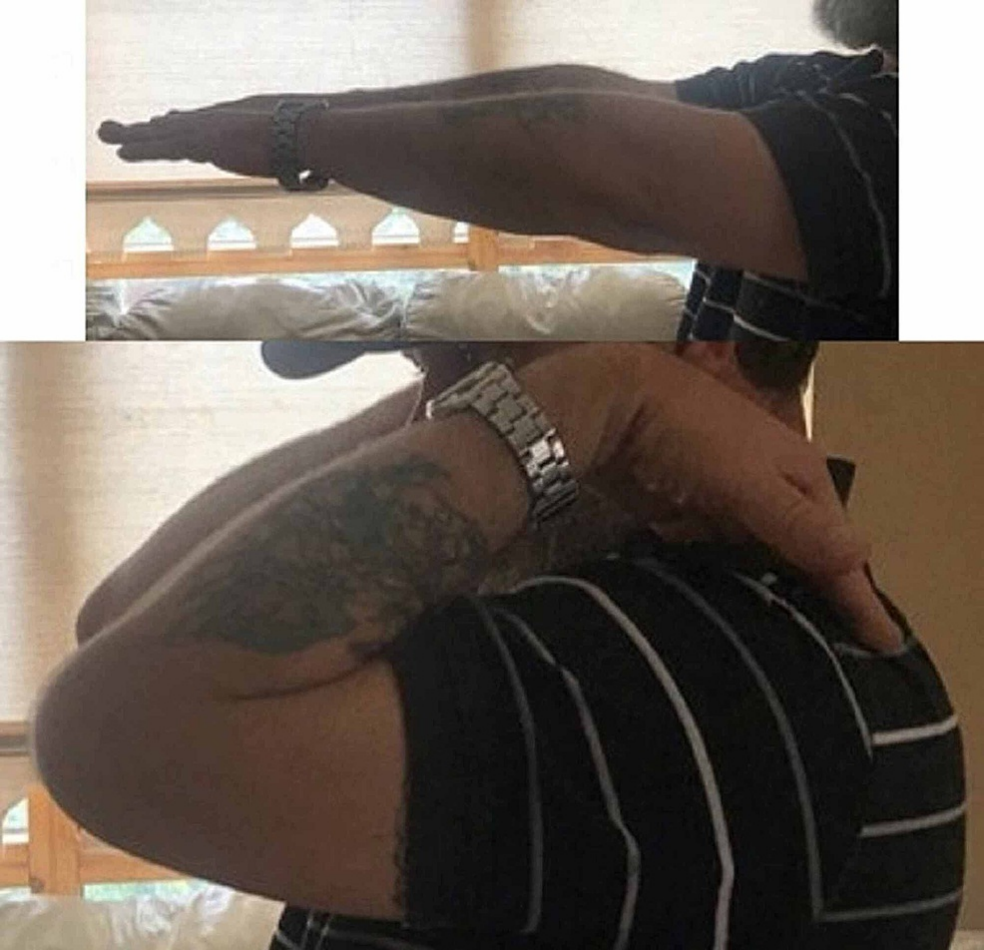
Photographs sent in by the patient, demonstrating his range of movement.

The incarcerated joint capsule was excised. The UCL and common flexor origin were repaired with suture anchors. Figure 4 shows the position of these anchors on postoperative X-ray. The ulnar nerve was stable through range of movement and so was not transposed. Postoperatively the patient was placed in an above elbow backslab for two weeks. After this he was allowed to mobilize freely and engaged in an intensive physiotherapy program. He has had no further episodes of instability and has returned to a full range of movement (Figure 5).

## Discussion

Anterior elbow dislocations without fracture are rare. Vijayan et al. carried out a literature review and published a case report on the topic in 2019 [5]. In total during their literature review only 21 cases were reported between 1922 and 2018. In 10 of these cases the method of treatment was not given. In five cases the management was a closed reduction and immobilization between five days and three weeks [6-9]. The remaining cases report some form of soft tissue repair and stabilization, with ulno-humeral k-wiring used in three of the cases.

Anterior elbow dislocations result in different soft tissue injury patterns compared to the more common posterior dislocations. O’Driscoll et al. describes how soft tissues are disrupted in a circular pattern from lateral to medial in posterior dislocations [10-11]. This is described as a “Circle of Horii” injury [12]. In the case we describe there was extensive soft tissue injury to the medial structures of the elbow, disruption of the anterior capsule, and no clinical or radiographic evidence of injury to the lateral structures. This suggests a pattern of injury opposite to the described circle of Horii, starting on the medial side and progressing laterally. It is also worth noting with the degree of injury to the medial side of the elbow it is important to be vigilant about the possibility of ulnar nerve injury. The clinical picture, Figure 3, included shows the degree of bruising and edema to the nerve in this case, though clinically there was no evidence of any deficit.

Bell et al. has described an algorithm for management of simple posterior elbow dislocations [13]. This algorithm is based on the stability of the elbow after reduction, as well as the functional demands of the patient, with a lower threshold for operative intervention in throwing athletes. Given the rarity of simple anterior elbow dislocations the rates of instability, stiffness, and pain are not known. While this algorithm may also be suitable in anterior dislocation cases there is no high level evidence in the literature on the topic.

## Conclusions

Given the rarity of the injury pattern, and the lack of evidence to guide treatment, we would recommend a cautious approach to these injuries with careful early evaluation of stability. Surgeons should have a low threshold for progressing to higher order imaging and EUA if there is any evidence of instability after initial reduction. CT will be more sensitive for picking up bony injuries, which may be missed on initial plan radiographs. MRI provides useful information on the soft tissue structures and allows you to anticipate which structures may be damaged and how you might repair them. The technique we used in this case allowed for early mobilization of the elbow and resulted in a good functional outcome.

We would advocate that providing enough stability to allow early mobilization is the key to success for any type of elbow dislocation. We report a rare variation of this common type of injury and hope that this report may be of use to other surgical teams dealing with similar presentations.
